# Interhospital transfer dynamics for patients with intracranial hemorrhage in Massachusetts

**DOI:** 10.3389/fneur.2024.1409713

**Published:** 2024-07-31

**Authors:** Ruchit V. Patel, Lilin Tong, Bradley J. Molyneaux, Nirav J. Patel, Mohammed A. Aziz-Sultan, Amar Dhand, Wenya Linda Bi

**Affiliations:** ^1^Department of Neurosurgery, Brigham and Women’s Hospital, Boston, MA, United States; ^2^Harvard Medical School, Boston, MA, United States; ^3^Boston University Aram V. Chobanian & Edward Avedisian School of Medicine, Boston, MA, United States; ^4^Department of Neurology, Brigham and Women’s Hospital, Boston, MA, United States

**Keywords:** healthcare systems, health infrastructure, intracranial hemorrhage, patient transfers, socioeconomics

## Abstract

**Introduction:**

Intracranial hemorrhages present across a spectrum of clinical phenotypes, with many patients transferred across hospitals to access higher levels of neurocritical care. We sought to characterize patient dispositions following intracranial hemorrhage and examine disparities associated with interhospital transfers.

**Methods:**

Using the Healthcare Cost and Utilization Project database, we mapped and identified factors influencing the likelihood of patient transfers and receipt of specialist interventional procedures following intracranial hemorrhage.

**Results:**

Of 11,660 patients with intracranial hemorrhage, 59.4% had non-traumatic and 87.5% single compartment bleeds. After presentation, about a quarter of patients were transferred to another facility either directly from the ED (23.0%) or after inpatient admission (1.8%). On unadjusted analysis, patients who were white, in the upper income quartiles, with private insurance, or resided in suburban areas were more frequently transferred. After adjusting for patient-and hospital-level variables, younger and non-white patients had higher odds of transfer. Hospital capabilities, residence location, insurance status, and prior therapeutic relationship remained as transfer predictors. Transferred patients had a similar hospital length of stay compared to admitted patients, with 43.1% having no recorded surgical or specialist interventional procedure after transfer.

**Discussion:**

Our analysis reveals opportunities for improvement in risk stratification guiding transfers, as well as structural challenges likely impacting transfer decisions.

## Introduction

Traumatic and non-traumatic intracranial hemorrhages encompass a broad spectrum of presentations, risk, and clinical sequelae (1, 2). For example, they can range anywhere from small traumatic sulcal subarachnoid bleeds which require minimal follow-up to clinically impactful non-traumatic aneurysmal subarachnoid hemorrhages that necessitate urgent evaluation. However, they are frequently labeled under a singular label of “head bleed” in determination of disposition after initial presentation. Depending on where patients with intracranial hemorrhage initially present, whether local non-tertiary care facilities to regional trauma or primary stroke centers, significant variability exists in their subsequent disposition and management.

A substantial proportion of these patients undergo interhospital transfer from the original presenting facility to access higher level specialty neurocritical and neurosurgical care (3, 4). This reflects the potentially grave clinical consequences with delay in care for pathologies which may need urgent operative intervention. Indeed, advanced diagnostic resources, surgical capabilities, and expertise in intracranial hemorrhage management are thought to positively impact outcomes for critically ill patients (5–8). However, while some studies linked transfers to lower in-patient mortality, others demonstrate no difference, with one study even recording worse cognitive outcomes in transferred patients (4, 9, 10). Additionally, there are financial and social strains in transferring patients which are weighed against the likelihood of benefit from an interhospital transfer (11, 12).

Given the variability in the natural history of intracranial hemorrhages, it is likely there are optimal scenarios and patient presentations following intracranial hemorrhage that would benefit from facility transfers and escalation of care while other patients with minor bleeds who are at low likelihood of clinical sequelae may be better monitored locally. We examined patterns and possible disparities in intracranial hemorrhage associated patient transfers using inpatient and emergency department insurance data from hospitals across Massachusetts through the Healthcare Cost and Utilization Project (HCUP) (13, 14). Through retrospective analysis, we explored hospital and patient-level factors for head bleed transfers and the clinical consequences after transfer. Dissecting the components influencing interhospital transfers is an important step toward creating intracranial hemorrhage management pathways that provide efficient, equitable, and effective care.

## Methods

### Patient population identification

We identified all patients with intracranial hemorrhage encountered in an emergency room or inpatient facility in Massachusetts in 2018 and 2019 using the HCUP State Emergency Department Database (SEDD) and State Inpatient Database (SID). HCUP was created and maintained by the Agency for Healthcare Research and Quality (AHRQ), which compiles state and national emergency department and inpatient discharge data. To capture the characteristics of facilities that patients presented to, we incorporated data from three additional sources: (1) American Hospital Association Annual Survey of Hospitals (2012), (2) Area Deprivation Index (ADI) from the Neighborhood Atlas, and (3) hospital capability data from publicly available institutional material (15, 16). As HCUP databases are limited datasets, this study was not subject to Institutional Review Board approval under the Health Insurance Portability and Accountability Act. Individual patients are not identifiable as part of the limited dataset and as an administrative database, informed consent was managed by HCUP. This analysis was approved under the Data Use Agreement (DUA) with HCUP, and all methods were carried out in accordance with HCUP DUA guidelines.

SEDD and SID patient claims were linked and ordered chronologically using a patient identifier (‘VisitLink’) and a timing variable (‘DaystoEvent’). To identify patients with non-traumatic and traumatic intracranial hemorrhage, we extracted all patient encounters with an ICD-10-CM code corresponding to intracranial hemorrhage (S06.4, S06.5, S06.6, I60, I61, I62). ICD-10-CM codes were selected based on literature validation (17).

We defined four different disposition routes for patients with intracranial hemorrhage to depart an ED: (1) direct discharge from the ED, (2) admission to the same hospital facility, (3) transfer from the ED to a different inpatient hospital facility (ED transfer), and (4) admission to the same hospital facility before transfer to another inpatient facility (inpatient transfer). Patients were categorized as admitted to the same hospital facility (route 2) if the source of admission was the ED and there were no claims generated at a separate facility. Patients were categorized as undergoing an ED transfer (route 3) if the disposition from the ED encounter indicated “transfer to other facility” and there was an inpatient record for the same patient within 1 day of the ED discharge record. We chose 1 day as the cutoff as most ED to inpatient transfers occur within hours of being formally discharged from the ED. This further helps distinguish between patients who were transferred versus those who had a separate inpatient admission (18). Finally, patients were categorized as undergoing an inpatient transfer (route 4) if an inpatient claim was followed by a transfer-specific second inpatient claim at a different facility within 1 day of discharge.

### Patient and hospital variables

Patient presentations were analyzed using ICD-10-CM codes from inpatient claims if available, as inpatient data were more robustly coded compared to ED data. Hemorrhages were categorized based on the mode of injury (traumatic, non-traumatic), bleed compartment (epidural, subdural, subarachnoid, intraparenchymal, unspecified), and the number of compartments affected (single, multiple). Patient-level characteristics including age, sex, race, comorbidities (Elixhauser comorbidity score), socioeconomic status, and insurance were extracted (19). Hospital-level variables encompassed facility capabilities (presence of neurosurgery and/or intensive care), teaching status, location-based Area Deprivation Index (ADI), number of beds, trauma level status, and prior patient visits to the same hospital or integrated delivery network.

To understand outcomes following patient transfers, we quantified interventions performed on admitted versus transferred patients. Interventions included intracranial access (e.g., craniotomy, ventricular drain), endotracheal intubation, and venous central line placement. Further, we compiled the inpatient length of stay (LOS) of based on transfer status.

### Data analysis

Patient and hospital characteristics were compared between admitted and transferred patients using the Student’s t-test, Wilcoxon rank sum test, and Chi-squared test with post-hoc pairwise comparison using the Tukey test. To determine patient and hospital level predictors of transfer following intracranial hemorrhage, we performed a univariate and multivariate logistic regression, adjusting for patient level (type of intracranial bleed, traumatic versus non-traumatic presentation, age, sex, race, insurance payer, Elixhauser comorbidity score, metropolitan location, income quartile) and hospital level variables (hospital trauma center level, teaching status, neurosurgical capability, intensive care capability, teaching status, area deprivation index, prior therapeutic relationship with hospital, and prior therapeutic relationship within the integrated delivery network).

The frequency of interventional procedures (intracranial, intubation, and central line) was compared using the Chi-squared test. Predictors for interventional procedures for transferred versus admitted patients were determined through a multivariate logistic regression, controlling for type of intracranial bleed, traumatic versus non-traumatic presentation, age, sex, race, Elixhauser comorbidity score, metropolitan location, income quartile, and hospital trauma center level. Additional hospital level variables such as neurosurgical capability and teaching status were not adjusted for in the multivariate regression after removal through a backwards elimination approach. For all statistical tests, variables were considered significant at *p* < 0.05.

## Results

### Patient presentation

**Figure 1:**
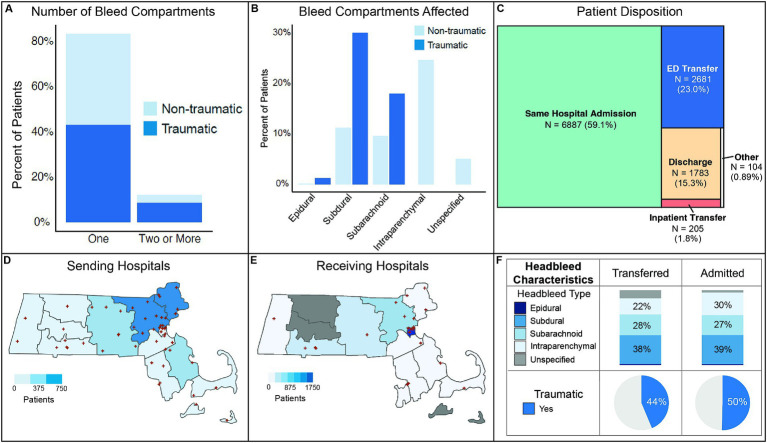
Overview of patient cohort and transfer network. **(A)** Number of compartments affected by traumatic and non-traumatic intracranial hemorrhage. **(B)** Intracranial hemorrhage prevalence by bleed compartment. **(C)** Patient disposition following initial hospital presentation. **(D)** Locations and patient sending frequency of hospitals in Massachusetts. **(E)** Locations and patient receiving frequency of hospitals in Massachusetts. **(F)** Distribution of intracranial hemorrhage characteristics and trauma status for transferred versus admitted patients.

We identified 11,660 patients in 2018 and 2019 across Massachusetts who presented with an intracranial hemorrhage. 59.4% of patients presented with non-traumatic intracranial hemorrhage versus 40.6% presented with traumatic intracranial hemorrhage. Of the patients with intracranial hemorrhage compartment(s) specified, 87.5% of bleeds localized to only one compartment while 12.5% affected multiple compartments. Single compartment intracranial hemorrhage split between traumatic (51.8%) and non-traumatic (48.2%) while multi-compartment intracranial hemorrhage largely stemmed from traumatic etiologies (70.7%, Figure 1A). A primary intracranial bleed compartment was designated for each patient. Subdural hematomas predominated (41.3%), followed by subarachnoid (27.6%), intraparenchymal (24.6%) and epidural (1.4%) bleeds, with 5.1% patients having an unspecified bleed type (Figure 1B).

### Disposition

Following presentation to an initial ED, 59.1% of patients were admitted to the same inpatient facility (Figure 1C). Approximately a quarter of patients underwent interhospital transfer, with 23.0% having an ED transfer and 1.8% an inpatient transfer after admission. 15.3% of patients were discharged directly from the ED.

Counties with lower population densities in Massachusetts were more likely to transfer intracranial hemorrhage patients while more dense population and medical facility areas more frequently received patients (Figures 1D,E). Though a slightly greater proportion of admitted patients had intraparenchymal bleeds compared to transferred patients, bleed compartments and traumatic mechanism were largely similar between admitted and transferred patients (Figure 1F).

**Figure 2:**
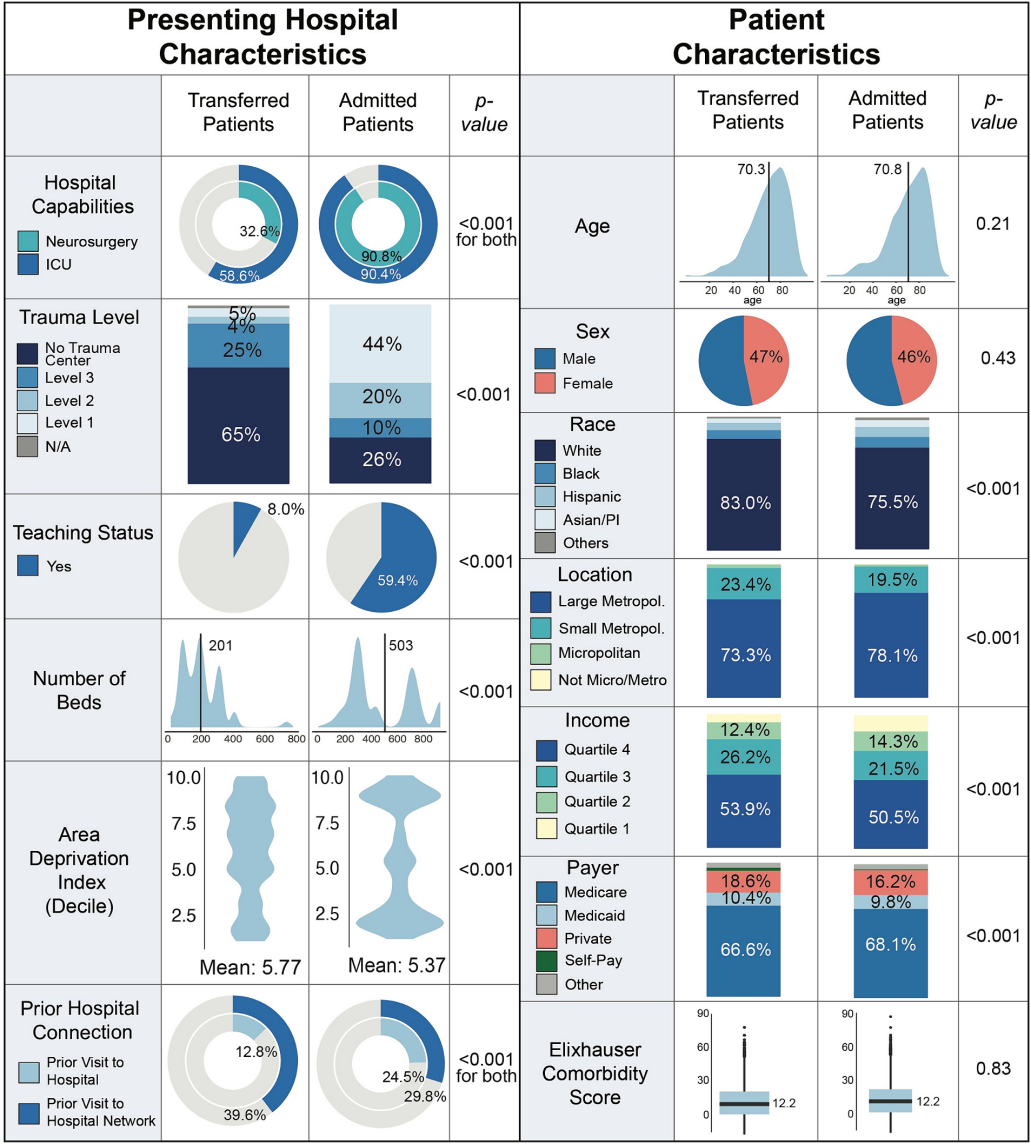
Patient and hospital level characteristics stratified by transfer versus admitted status. ADI, area deprivation index.

Across both admitted and transferred patients, a majority of patients presented to a hospital or an integrated hospital network with which they had no prior therapeutic relationship (Figure 2). However, compared to transferred patients, admitted patients more frequently had a prior therapeutic relationship with the same presenting hospital (24.5% vs. 12.8%, *p* < 0.001). Admitting hospitals enriched for Level 1 or 2 trauma centers (Level 1: 44% vs. 5%, Level 2: 20% vs. 4%, *p* < 0.001) and teaching status (59.4% vs. 8.0%). While admitting hospitals were on average larger than transferring hospitals (503 vs. 201 patient beds), almost half of these admitting facilities had a low number of patient beds more closely resembling transferring hospitals. Admitting hospitals had significantly more neurosurgical and intensive care unit (ICU) capabilities compared to transferring hospitals (neurosurgery: 90.8% versus 32.6%, ICU: 90.4% vs. 58.6%, *p* < 0.001). Additionally, though admitting hospitals were on average located in better resourced settings compared to transferring hospitals (admitting ADI: 5.37 vs. transferring ADI: 5.77, *p* < 0.001), about half of admitting hospitals were located in lower resourced settings.

Patients who were admitted versus those who were transferred harbored similar comorbidity scores on average (*p* = 0.83; Figure 2). A greater proportion of transferred patients were white, in the top two income quartiles, and had private insurance (all *p* < 0.001). Admitted patients more commonly presented from larger metropolitan areas (78.1% admitted vs. 73.3% transferred) while transferred patients more commonly resided in smaller metropolitan and suburban areas (19.5% admitted vs. 23.4% transferred, *p* < 0.001).

### Factors influencing patient transfer

On univariate analysis, patients were more likely to be transferred if they were white, had private insurance, or if they presented from smaller metropolitan or micropolitan regions (all *p* < 0.01, Table 1). Prior therapeutic relationship influenced transfers; patients were more likely to be transferred if they presented within a previously visited integrated hospital network but were less likely to be transferred if they presented to a previously visited hospital (both *p* < 0.05). Patients were also less likely to be transferred if they presented to hospitals with a higher overall resource status (lower ADI, *p* < 0.01). Further, those in the lower two income quartiles, with intraparenchymal bleeds (ref: epidural), or with traumatic bleeds (ref: non-traumatic) were less likely to be transferred (*p* < 0.01). As expected, transfer was also less likely if patients presented to a Level 1 or 2 trauma center, teaching hospitals, and facilities with neurosurgery and/or ICU capabilities (*p* < 0.001).

**Table 1:** Univariate predictors of hospital transfers after presenting with an intracranial hemorrhage.

Variable	OR	95% CI	*p*
**Bleed compartment (Ref: Epidural)**			
Subdural	0.86	0.62–1.20	ns
Subarachnoid	0.91	0.65–1.28	ns
Intraparenchymal	0.64	0.45–0.90	<0.001
Traumatic Bleed	0.75	0.69–0.81	<0.001
**Age (Ref: <65)**			
65–75	1.09	0.97–1.21	ns
>75	0.94	0.86–1.03	ns
Sex (Female)	1.03	0.95–1.11	ns
**Race (Ref: White)**			
Black	0.77	0.65–0.90	<0.001
Hispanic	0.72	0.61–0.85	<0.001
Asian/Pacific islander	0.64	0.51–0.80	<0.001
Other	0.71	0.50–0.99	ns
**Insurance payer (Ref: Medicare)**			
Medicaid	1.08	0.94–1.23	ns
Private	1.17	1.05–1.30	<0.01
Self-Pay	1.30	1.19–1.44	<0.001
Elixhauser comorbidity score	1.01	0.99–1.01	ns
**Patient presenting location (Ref: Large metropolitan area)**			
Small metropolitan area	1.19	1.08–1.31	<0.001
Micropolitan area	1.42	1.11–2.81	<0.01
**Income quartile (Ref: Q4: >82 k)**			
Q1: 0-48 k	0.54	0.46–0.63	<0.001
Q2: 48 k-60 k	0.82	0.73–0.93	<0.001
Q3: 60 k-82 k	1.08	0.98–1.19	ns
**Trauma level (Ref: No status)**			
Level 1	0.03	0.03–0.04	<0.001
Level 2	0.06	0.05–0.08	<0.001
Level 3	0.73	0.65–0.82	<0.001
Neurosurgical capability (Yes)	0.05	0.04–0.05	<0.001
ICU capability (Yes)	0.15	0.14–0.17	<0.001
Teaching status (Yes)	0.04	0.04–0.05	<0.001
Bed number	0.99	0.99–1.00	ns
ADI decile	1.06	1.05–1.07	<0.001
Previous visit to same hospital (Yes)	0.17	0.15–0.19	<0.001
Previous visit to same IDN (Yes)	1.41	1.29–1.53	<0.001

ICU, intensive care unit; IDN, integrated delivery network.

**Figure 3:**
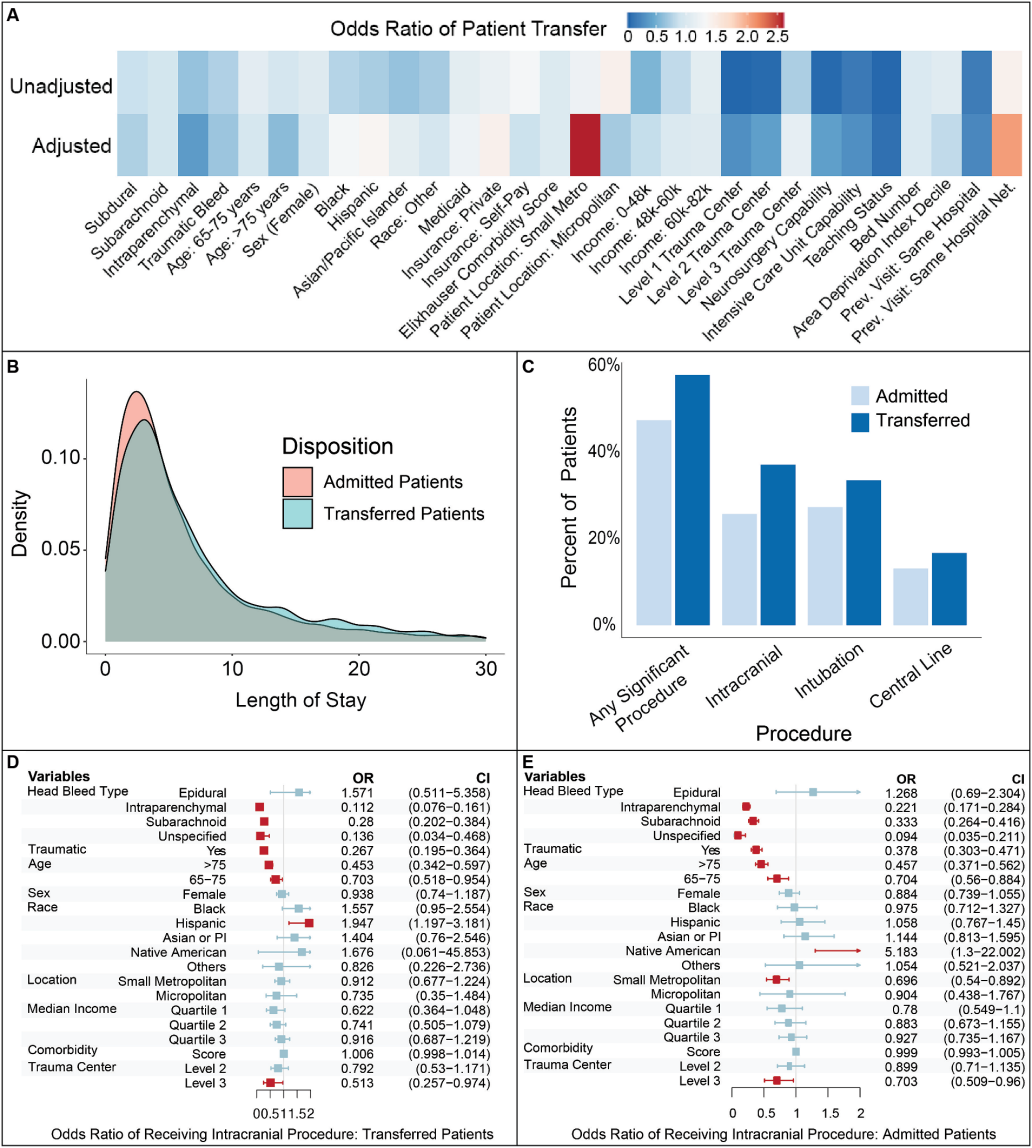
Analysis of factors influencing patient transfers and procedures following transfer. **(A)**. Comparison of unadjusted and adjusted odds ratios for patient transfers following intracranial hemorrhage. **(B)**. Frequency of interventional procedures for transferred versus admitted patients. **(C)**. Distribution of hospital length for transferred versus admitted patients. **(D)**. Multivariate adjusted odds ratio of receiving an intracranial procedure following transfer. **(E)**. Multivariate adjusted odds ratio of receiving an intracranial procedure for patients admitted with intracranial hemorrhage. Red shading indicates odds ratios significant at *p* < 0.05. Prev.: previous, Net.: network.

After multivariate adjustment, hospital-level variables predominated over patient-level factors in predicting the likelihood of patient transfers (Table 2; Figure 3A). Patients who presented to Level 1 or 2 trauma centers, facilities with neurosurgery and/or ICU capabilities, and teaching hospitals had lower likelihood of transfer (all *p* < 0.001). Prior therapeutic relationship remained a strong modifying factor, with patients significantly less likely to be transferred if they presented to a hospital which they previously visited (*p* < 0.001). Intraparenchymal (ref: epidural) as well as traumatic bleeds also continued to show a lower likelihood of transfer (both *p* < 0.01).

**Table 2:** Multivariate adjusted predictors of hospital transfers after presenting with an intracranial hemorrhage.

Variable	OR	95% CI	*p*
**Bleed compartment (Ref: Epidural)**			
Subdural	0.74	0.43–1.29	0.295
Subarachnoid	0.91	0.52–1.59	0.744
Intraparenchymal	0.38	0.21–0.68	0.001
Traumatic bleed	0.67	0.56–0.80	<0.001
**Age (Ref: <65)**			
65–75	0.95	0.76–1.18	0.634
>75	0.59	0.48–0.73	<0.001
Sex (Female)	0.94	0.83–1.07	0.369
**Race (Ref: White)**			
Black	1.29	0.96–1.72	0.089
Hispanic	1.36	1.01–1.83	0.042
Asian/Pacific Islander	1.08	0.75–1.54	0.673
Other	0.93	0.55–1.50	0.795
**Insurance payer (Ref: Medicare)**			
Medicaid	1.21	0.92–1.60	0.169
Private	1.42	1.15–1.77	0.001
Self-pay	0.87	0.44–1.66	0.670
Elixhauser comorbidity score	1.01	1.00–1.01	0.002
**Patient presenting location (Ref: Large metropolitan area)**			
Small metropolitan area	2.61	2.10–3.25	<0.001
Micropolitan area	0.70	0.42–1.22	0.192
**Income Quartile (Ref: Q4: >82 k)**			
Q1: 0-48 k	0.84	0.64–1.11	0.251
Q2: 48 k-60 k	1.00	0.81–1.25	0.133
Q3: 60 k-82 k	1.04	0.88–1.23	0.231
**Trauma level (Ref: No status)**			
Level 1	0.30	0.23–0.40	<0.001
Level 2	0.40	0.31–0.52	<0.001
Level 3	1.11	0.93–1.33	0.257
Neurosurgical capability (Yes)	0.40	0.34–0.47	<0.001
ICU capability (Yes)	0.30	0.25–0.36	<0.001
Teaching status (Yes)	0.09	0.07–0.12	<0.001
Bed number	0.99	0.99–1.00	<0.001
ADI decile	0.82	0.79–0.84	<0.001
Previous visit to same hospital (Yes)	0.25	0.21–0.30	<0.001
Previous visit to same IDN (Yes)	2.08	1.79–2.42	<0.001

ICU, intensive care unit; IDN, integrated delivery network.

Multivariate correction also revealed certain co-dependent patient and hospital level predictors, different from what was seen in unadjusted univariate analysis. Presentation to poorer-resourced hospitals and patient age greater than 75 years old resulted in a lower likelihood of transfer (both *p* < 0.001). The impact of race also inverted after adjusting for mutually dependent factors: Hispanic patients had a significantly greater likelihood of transfer (*p* < 0.05) while Black patients had a trend toward a greater likelihood of transfer (*p* = 0.09) compared to white patients. Income quartile was no longer a significant predictor for transfer.

Controlling for patient- and hospital-level variables also showed the outsized impact of where patients live, prior therapeutic relationship within a hospital network, and insurance status. Patients who resided in smaller metropolitan areas had more than two times the odds of transfer compared to those from large metropolitan areas (*p* < 0.001). Previous visits within the same hospital network resulted in a similarly increased twofold odds of transfer (*p* < 0.001). Patients with private insurance also had a significantly increased likelihood of transfer, much higher than the odds ratio seen in univariate analysis (*p* = 0.001).

### Outcomes after transfer

Admitted and transferred patients exhibited a similar hospital length of stay (LOS) (Figure 3B). Median and average length of stay did not differ between directly admitted versus transferred patients (median: 4 vs. 5 days, average: 7.42 vs. 7.92 days, *p* > 0.05), with 23.1% of patients discharging within the first 2 days after transfer. Both distributions were skewed right, with admitted patients having an LOS range from 1 to 255 days and transferred patients having a range from 1 to 96 days. There was no significant difference in inpatient mortality between admitted and transferred patients (OR = 0.99, 95%CI = 0.78–1.23), even after adjusting for patient-level factors (Supplementary Figure S1).

Transferred patients were more likely to undergo surgical or specialist procedures compared to admitted patients (57.9% vs. 47.4%, *p* < 0.01, Figure 3C). This trend was consistent across major procedure subcategories, including intracranial access (37.1% transferred vs. 25.7% admitted patients), intubation (33.5% transferred vs. 27.3% admitted patients), and central line placement (16.7% transferred vs. 13.1% admitted patients). Conversely, nearly half of patients received no significant invasive procedure following their transfer.

Patients older than 65 years and those transferred to a Level 3 trauma center were significantly less likely to receive intracranial intervention, after multivariate adjustment (*p* < 0.01; Figure 3D). Intraparenchymal and subarachnoid bleeds were less likely to necessitate an intervention compared to subdural hematomas (*p* < 0.01). Traumatic bleeds were also less likely to receive intervention compared to non-traumatic bleeds (*p* < 0.01). Similar odds for intracranial intervention were observed for admitted patients, indicating relative consistency in management of intracranial hemorrhage whether patients are directly admitted or transferred (Figure 3E).

## Discussion

The burden of traumatic and non-traumatic intracranial hemorrhage is high in the United States, with a trend toward increasing incidence over the past two decades (20, 21). Through a multicenter statewide approach, we show a significant proportion of patients with intracranial hemorrhage are transferred between hospitals. This demonstrates the presence of a robust transfer network across health systems in Massachusetts, enabling timely access to high levels of neurocritical and neurovascular care. While care is being escalated, the heterogeneity in patients with intracranial hemorrhage, rising cost of healthcare delivery, and limitations in hospital capacity make it critical to understand appropriate transfer scenarios, presence of inequities, and potential opportunities to improve efficiency of healthcare delivery without compromising quality. Our analysis highlights specific hospital and patient characteristics which may influence transfer, and intracranial hemorrhage phenotypes which may have more variable benefits from an interhospital transfer.

Patient age, race, and income impacted transfer in a nuanced fashion, demonstrating interdependence with other system factors. In univariate analysis, non-White patients, those in lower income quartiles, or those on public insurance had lower odds of transfer. However, after adjustment across patient and hospital variables, income became a non-significant feature. Instead, patient age, location of presentation, insurance status, hospital capabilities, and prior therapeutic relationships to hospitals/hospital networks strengthened as significant modifiers. In addition, the impact of race inverted. Non-White patients, including Hispanic and Black patients, had a higher adjusted likelihood of transfer. These findings are congruent with recent studies examining transfers for acute ischemic stroke care where patients of color had higher odds of transfer after adjustment for hospital level variables and insurance status (23, 24). This suggests that differential transfers by race may reflect other systemic intersectional variables such as being on public insurance, presentation to poorer-resourced hospitals, or prior visits to the same hospital. It is crucial to recognize and address the structural inequities between hospitals when developing and evaluating transfer practices and protocols.

Hospitals that preferentially admit brain bleed patients largely reflected the paradigm of escalation of care for intracranial hemorrhage. Many of these facilities were tertiary-care, academic medical centers with advanced subspecialty neurosurgical and critical care. We also identified a significant number of smaller medical facilities which admitted rather than transferred intracranial hemorrhage patients. These admitting facilities were not Level 1–2 trauma centers (36%), were not of teaching status (41%), and did not have neurosurgery or intensive care capabilities (approximately 10%). Further, these hospitals had a significantly lower number of beds compared to larger admitting hospitals and were less resourced per their ADI. Interestingly, the characteristics of smaller admitting hospitals matched those of transferring hospitals, with two possible implications. One possibility is that these smaller admitting hospitals have developed a structure of effectively managing intracranial hemorrhage patients even without the capabilities of larger facilities. Alternatively, it may be that certain smaller admitting facilities are be unable to consistently transfer their patients and therefore treat on-site (22). However, based on our findings, we found no difference in inpatient mortality between admitted and transferred patients, indicating that direct admissions from smaller hospitals were likely being made appropriately.

The lack of listed interventional procedures on more than 40% of intracranial hemorrhage patients following interhospital transfer reveals opportunities for improvement in risk stratification and transfer decisions. While medical treatment is a significant component of intracranial hemorrhage management and not captured by this dataset, there are likely patients being transferred for surgical evaluation who do not need or receive any intracranial intervention. Almost a quarter of transferred patients were discharged within 2 days. Shorter lengths of stay could reflect a population of patients who benefitted from escalation care but may also indicate patients for whom transfer was not required. Additionally, there were a slightly greater number of transferred patients compared to admitted patients who remained hospitalized greater than 20 days (in the tail of the LOS distribution). This might be a population of patients who were appropriately transferred and required significant procedures, or were medically complex with limited safe discharge options (25). Given the inpatient resources allocated to intracranial hemorrhage management, understanding the unique medical needs of these transfer patient sub-populations may help with hospital capacity allocation and resource utilization. These observations broadly demonstrate the need to identify patients who can maximally benefit from a transfer and calls for creation of established clinical pathways to guide triage of transfers.

When assessing adjusted predictors for transfers and intracranial procedures, there were trends across bleed types and patient populations. Intraparenchymal bleeds had lower odds for transfer compared to epidural bleeds. This likely reflects the natural history of intraparenchymal bleeds, where many bleeds are small and aggressive immediate management focuses on reversal of anticoagulation and control of blood/intracranial pressure, rather than escalation to another facility (26–28). Following transfer, intraparenchymal and subarachnoid bleeds were less likely to undergo an intracranial procedure compared to subdural bleeds. While we were limited in our assessment of traumatic intraparenchymal bleeds given HCUP data availability, this is in line with current practice standards for non-traumatic intraparenchymal bleeds and non-operative management of certain subarachnoid etiologies (29, 30). Traumatic bleeds had both a lower likelihood of transfer and intracranial intervention which may be a result of several scenarios. In high-velocity traumas, patient stability, clinical decompensation, and the need for non-neurosurgical intervention might influence the ability to transfer. In low-velocity traumas, small bleeds might not require escalation of care or an operation. The lower likelihood of intervention in patients older than 65 further shows the nuanced role of demographics and bleed characteristics in patient selection for intracranial procedures. Taken together, the heterogeneity in transfer predictors across intracranial bleed types likely necessitates deeper clinical investigation into bleed specific management challenges.

This study faced several limitations, some of which were linked to using an administrative insurance claims dataset. Inaccurate ICD or patient demographic coding could have impacted the underlying data analyzed (31). As claims data is based on standardized data entry, we were unable to capture more granular data on intracranial hemorrhage patient presentation. This included acute clinical concerns, patient decompensation, and facility status (e.g., capacity disaster, reduced staffing) that could have influenced transfer decisions. Similarly, we did not have access to exact indications for transfer. Our analysis of post-transfer outcomes was also subject to variability in ICD coding for procedures and limited given the lack of robust mortality data. Nevertheless, our analysis uniquely highlights the dependency between predictors of intracranial hemorrhage patient transfers while identifying distinct sub-populations of hospitals and patients that can improve intracranial hemorrhage transfer decision making.

## Data availability statement

The data analyzed in this study is subject to the following licenses/restrictions: datasets are available following approved data use agreement with HCUP. Requests to access these datasets should be directed to https://hcup-us.ahrq.gov/databases.jsp.

## Author contributions

RP: Conceptualization, Data curation, Formal analysis, Investigation, Methodology, Visualization, Writing – original draft, Writing – review & editing. LT: Conceptualization, Data curation, Formal analysis, Investigation, Methodology, Visualization, Writing – original draft, Writing – review & editing. BM: Writing – original draft, Writing – review & editing. NP: Writing – original draft, Writing – review & editing. MA-S: Writing – original draft, Writing – review & editing. AD: Conceptualization, Methodology, Writing – original draft, Writing – review & editing. WB: Conceptualization, Data curation, Investigation, Methodology, Visualization, Writing – original draft, Writing – review & editing.

## Ethics statement

Ethical approval was not required for the study involving humans in accordance with the local legislation and institutional requirements. Written informed consent to participate in this study was not required from the participants or the participants’ legal guardians/next of kin in accordance with the national legislation and the institutional requirements.

## Supplementary material

Supplementary Figure S1 Comparison of inpatient mortality risk between transferred and admitted patients. Adjusted by type of bleed, presence of trauma, age, sex, insurance, comorbidity (calculated by Elixhauser Comorbidity Score), Income quartile by zip, and race.
