# Supplementary figures and images for: Interhospital transfer dynamics for patients with intracranial hemorrhage in Massachusetts
Source: Front Neurol. 2024 Jul 31;15:1409713. doi: 10.3389/fneur.2024.1409713 (PMC11322084; doi:10.3389/fneur.2024.1409713)

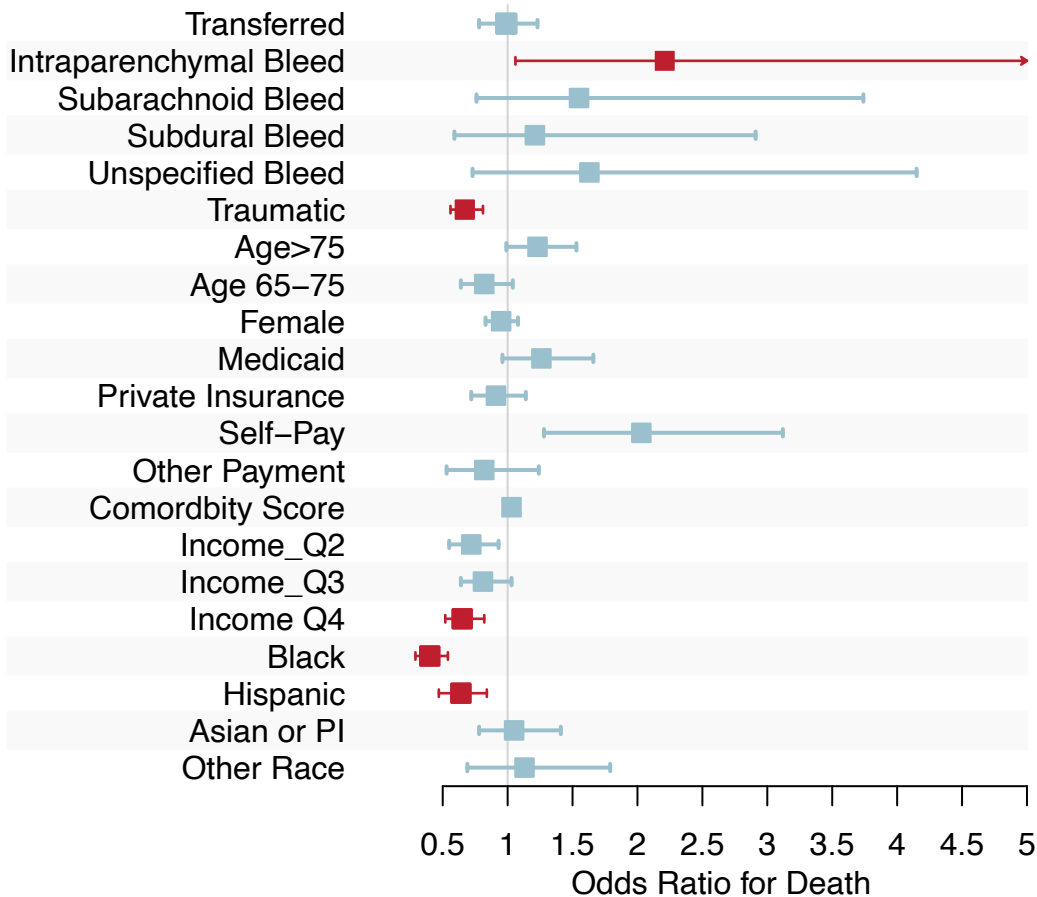

Supplement: Supplementary Figure S1 — Comparison of inpatient mortality risk between transferred and admitted patients. Adjusted by type of bleed, presence of trauma, age, sex, insurance, comorbidity (calculated by Elixhauser Comorbidity Score), Income quartile by zip, and race. [file Image_1.pdf]
